# Fabrication and Characterization of Xanthan Gum-cl-poly(acrylamide-co-alginic acid) Hydrogel for Adsorption of Cadmium Ions from Aqueous Medium

**DOI:** 10.3390/gels8010023

**Published:** 2021-12-27

**Authors:** Gaurav Sharma, Amit Kumar, Ayman A. Ghfar, Alberto García-Peñas, Mu. Naushad, Florian J. Stadler

**Affiliations:** 1 College of Materials Science and Engineering, Shenzhen Key Laboratory of Polymer Science and Technology, Guangdong Research Center for Interfacial Engineering of Functional Materials, Nanshan District Key Laboratory for Biopolymers and Safety Evaluation, Shenzhen University, Shenzhen 518060, China; mittuchem83@gmail.com (A.K.); fjstadlr@szu.edu.cn (F.J.S.); 2 International Research Centre of Nanotechnology for Himalayan Sustainability (IRCNHS), Shoolini University, Solan 173212, Himachal Pradesh, India; 3 School of Science and Technology, Glocal University, Saharanpur 247001, Uttar Pradesh, India; 4 Department of Chemistry, College of Science, King Saud University, P.O. Box 2455, Riyadh 11451, Saudi Arabia; aghafr@ksu.edu.sa; 5 Departamento de Ciencia e Ingeniería de Materiales e Ingeniería Química (IAAB), Universidad Carlos III de Madrid, Leganés, 28911 Madrid, Spain; alberto.garcia.penas@uc3m.es

**Keywords:** xanthum gum, polyacrylamide, cadmium ions, adsorption, Langmuir, Freundlich

## Abstract

The present research demonstrates the facile fabrication of xanthan gum-cl-poly(acrylamide-co-alginic acid) (XG-cl-poly(AAm-co-AA)) hydrogel by employing microwave-assisted copolymerization. Simultaneous copolymerization of acrylamide (AAm) and alginic acid (AA) onto xanthan gum (XG) was carried out. Different samples were fabricated by changing the concentrations of AAm and AA. A sample with maximum swelling percentage was chosen for adsorption experiments. The structural and functional characteristics of synthesized hydrogel were elucidated using diverse characterization tools. Adsorption performance of XG-cl-poly(AAm-co-AA) hydrogel was investigated for the removal of noxious cadmium (Cd(II)) ions using batch adsorption from the aqueous system, various reaction parameters optimized include pH, contact time, temperature, and concentration of Cd(II) ions and temperature. The maximum adsorption was achieved at optimal pH 7, contact time 180 min, temperature 35 °C and cadmium ion centration of 10 mg·L^−1^. The XG-cl-poly(AAm-co-AA) hydrogel unveiled a very high adsorption potential, and its adsorption capacities considered based on the Langmuir isotherm for Cd(II) ions was 125 mg·g^−1^ at 35 °C. The Cd(II) ions adsorption data fitted nicely to the Freundlich isotherm and pseudo-first-order model. The reusability investigation demonstrated that hydrogel retained its adsorption capacity even after several uses without significant loss.

## 1. Introduction

Wastewater remediation is attaining importance in the present era due to the severe scarcity of water in various countries. Various types of contaminants are added to the water bodies without any pretreatments such as dyes, heavy metal ions, pigments, pesticides, pharmaceutical effluents, personal care products, and radioactive wastes, etc. Among these, heavy metals are very common pollutants added from diverse sources such as electroplating, batteries manufacturing, metal treating in processing plants, sweltering of coal in power plants, incineration of petroleum, nuclear power stations, plastics, textiles, microelectronics, paper processing plants, and wood preservation, etc. [1,2,3].

Heavy metals are also known as trace elements as they are present in very less quantity few heavy metals such as iron, copper, chromium, zinc, magnesium, selenium, manganese and molybdenum, etc. are important and essential for several physiological and biochemical functions [4]. Whereas other heavy metals as arsenic, aluminum, lead, antinomy, mercury, barium, indium, beryllium, nickel, bismuth, cadmium, gold, gallium, germanium, lithium, platinum, tin, silver, titanium, strontium, tellurium, vanadium, thallium, and uranium do not have any such recognized physiological or biological functions and thus are non-essential [5,6,7,8]. Out of these heavy metals, cadmium is recorded as a very common contaminant added to groundwater and soil. Cadmium is extremely lethal and may threaten the aquatic ecosystem and humans. Acquaintance with cadmium for long periods of time can severely affect health and may result in diseases such as osteoporosis, cardiac failure, cancer and itai-itai disease, etc. [9,10]. The presence of cadmium in the environment has become a topic of concern, as it cannot be decayed by microorganisms, thus it unceasingly accumulates, transforms, and migrates in food chains. The cadmium bioavailability and migration in the ecosystem are influenced by its magnitude of adsorption with solid segments. The adsorption extent of cadmium ions depends upon its interaction between aqueous and solid phases and is entirely controlled by properties such as temperature, pH, surface area, ionic strength, and surface charge, etc. [11,12,13].

Numerous methods were implemented for remediation of noxious cadmium ions from wastewater these include phytoremediation, ion exchange, chemical precipitation, solvent extraction, coagulation, filtration, adsorption and membrane techniques, etc. [14,15]. As adsorption is a most effective and easy to handle technique it has been extensively used for heavy metals remediation. Numerous researchers are working on designing and fabricating highly efficient adsorbents which include modified activated carbon, zeolites, metal-organic frameworks, carbon nanotubes, biochar, MXenes, carbon nitride, and hydrogels, etc. [16,17,18,19]. Hydrogels are very efficient adsorbents as they possess greater functionalities, better swelling, good surface area and biocompatibility, etc. Diverse synthetic and natural polymer or gums have been used for the fabrication of superabsorbent hydrogel, these include chitosan, pectin, carrageenan, starch, gelatin, cellulose, chitin, sodium alginate, guar gum, gum arabica, tragacanth gum, xanthan gum, polyacrylamide (PAM), polyalginic acid, polyacrylic acid(PAA), poly(hydroxyethyl methacrylate) (PHEMA), Poly(glyceryl methacrylate) (PGMA), 1,1,1-trimethylolpropane trimethacrylate (TMPTMA), polyvinyl pyrrolidone (PVP), poly(ethylene glycol) dimethacrylate (PEGDMA), polymethacrylamide (PMAM), triethylene glycol dimethacrylate (TEGDMA), polyethylene glycol (PEG), polyvinyl alcohol (PVA), Poly(hydroxypropyl methacrylate) (PHPMA), and poly(ε-caprolactone) (PCL), etc. [20,21,22,23,24]. The high swelling ability of hydrogels helps in enriching the structure with high absorption and adsorption characteristics. When present in the aqueous solution, the polymer chains loosen up so as to swell more and more solvent inside it. Being eco-friendly in nature, hydrogels are now utilized for diverse applications ranging from water purification to biomedical. The crosslinking of monomers and polymer chains helps in enriching the structure with high swelling capacity, enhanced adsorption or absorption ability, and mechanical strength. 

So, herein, an efficient adsorbent was synthesized using xantham gum, acrylamide, and acrylic acid by a green method in which microwave radiations were used for the synthesis. The synthesized adsorbent hydrogel was used positively for the adsorption of Cd(II) ions from an aqueous solution. It is worth mentioning that the hydrogel swelling shoot up the adsorption process and lessened the needed time to reach 90% of optimum adsorption from 240 to 180 min. The adsorption process followed linear forms of the PSO and Freundlich model. The free energy of Cd(II) ions after adsorption disclosed the physical nature of adsorption, while PSO anticipated chemical interactions. Consequently, it can be established that the interactions are physiochemical in nature. 

## 2. Results and Discussion

The three different samples of XG-cl-poly(AAm-co-AA) hydrogel were prepared by varying the concentration of acrylamide and alginic acid, i.e., 1XG:1AAm:1 AA, 1XG:1AAm:0.5 AA, 1XG:1AAm:0.5 AA. The swelling experiments were performed for 36 hrs and swelling percentages were found to be 84%, 79%, and 96%, respectively. Thus sample with reactant ratio1XG:1AAm:0.5 AA was chosen for adsorption studies as it possesses the maximum swelling. The swelling test is the characteristic of any hydrogel, thus, the higher the swelling ability, the better the sorption activity will be.

**Figure 1:**
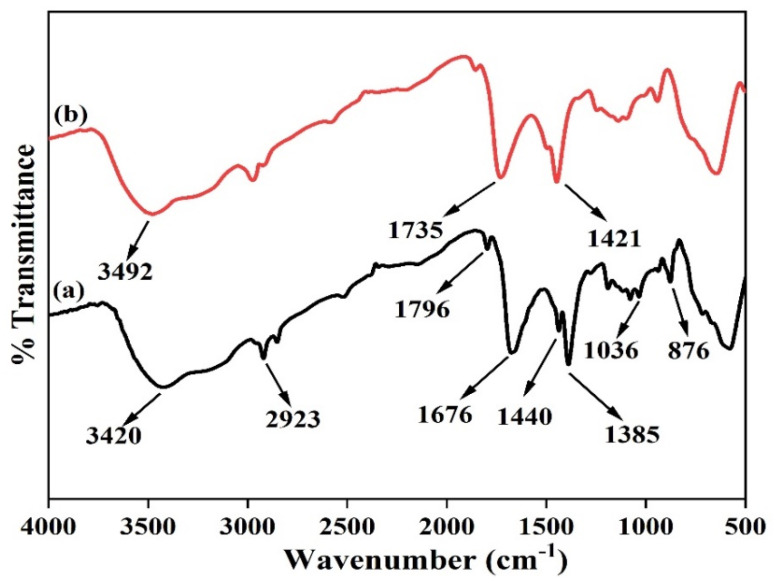
FTIR of spectra of xanthan gum-cl-poly(acrylamide-co-alginic acid) hydrogel (**a**) before adsorption and (**b**) after adsorption.

**Scheme 1:**
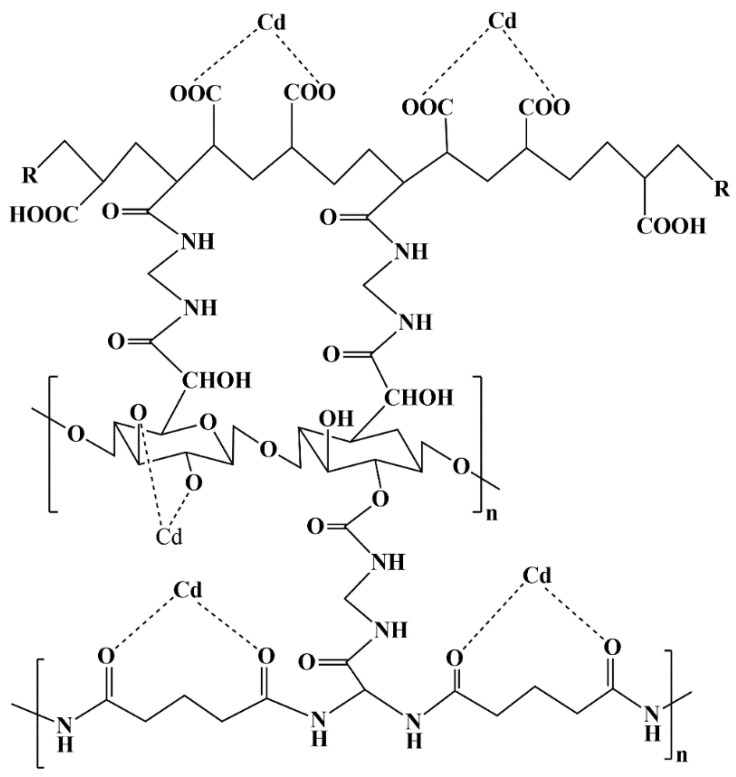
Probable scheme for the complex formation by the adsorption of Cd(II) ions onto XG-cl-poly(AAm-co-AA) hydrogel.

The results of FTIR spectra of XG-cl-poly(AAm-co-AA) hydrogel before and after Cd(II) ion adsorption are presented in Figure 1. The FTIR spectrum in Figure 1a represents the peaks obtained for XG-cl-poly(AAm-co-AA) hydrogel. It exhibits a prominent broad band at 3420 cm^−1^ signifying the stretching vibrations for the -OH groups of acrylic acid and xanthan gum [25]. The peak for the C-H stretching vibrations for the aliphatic units present in the hydrogel is detected at 2923 cm^−1^. The peaks at 1796 cm^−1^ and 1676 cm^−1^ are owing to the stretching of carbonyl groups and reveal symmetrical stretching for the carboxylate group [26]. Thus, these peaks are indicative of the C=O groups present in poly(acrylic acid), NMBA, and XG [27]. Another band at 1440 cm^−1^ can be ascertained to the −CH symmetric bending vibrations due to the existence of −CHOH− groups in the hydrogel [28]. The peak at 1385 cm^−1^ is due to the bending vibration of C-H of the isopropyl group, 1188 cm^−1^ is due to C-N stretching, 1124 cm^−1^ is owing to the -CO stretching, and 876 cm^−1^ and 714 cm^−1^ peaks can be consigned to N-H bending vibrations [29]. The band at 1036 cm^−1^ may possibly be owing to the stretching of the C-O bond of glycosidic bonds. Alike bands are present in the XG-cl-poly(AAm-co-AA) hydrogel after adsorption of Cd(II) ions with variation in peaks intensities and some additional shifts in the peaks. For example, the broad band observed at 3420 cm^−1^ in hydrogel was shifted to higher wavenumber 3492 cm^−1^, which could probably be due to the possible intermolecular interactions between the hydrogel and Cd(II) ions [30]. Furthermore, the characteristic peak of C=O at 1676 cm^−1^ was shifted to 1735 cm^−1^ suggesting the probable complexation of carbonyl units and Cd(II) ions. Thus, it can be concluded that the changes in the FTIR spectrum of hydrogel after Cd(II) ion adsorption is due to the physicochemical interactions. Considering the FTIR results, it can be concluded that the major groups that participated in the complex formation (Cd(II) adsorbed XG-cl-poly(AAm-co-AA) hydrogel), were –COOH, –C=O, and –OH groups. Thus, a probable scheme is presented (Scheme 1) that shows the expected structure of the complex formed.

**Figure 2:**
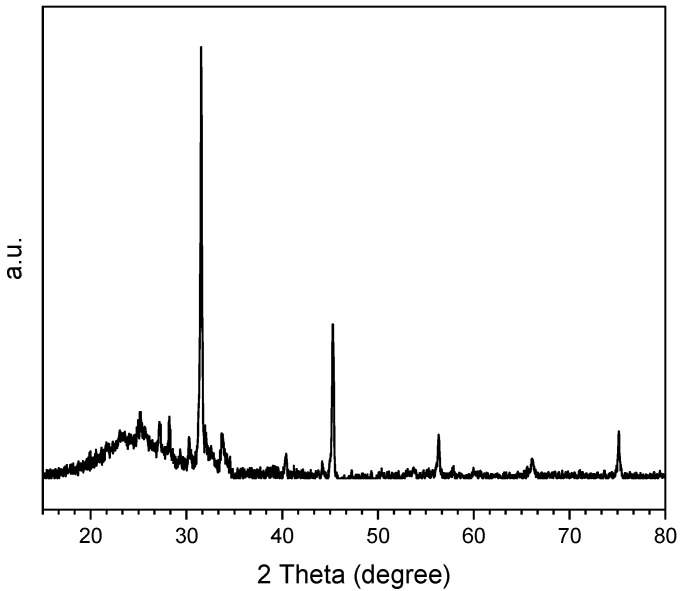
XRD of xanthan gum-cl-poly(acrylamide-co-alginic acid) hydrogel.

The X-ray diffractogram of XG-cl-poly(AAm-co-AA) hydrogel is shown in Figure 2. X-ray diffractometer PAN analytical X′Pert PRO was used during the study. The X-ray diffractometric examination was performed to disclose the phase (crystallinity or amorphous) of the XG-cl-poly(AAm-co-AA) hydrogel. Figure 2 depicts that the diffractogram of hydrogel was found to be semi-crystalline in nature. The previous studies of crude xanthan gum diffractogram show XRD peaks at a 2θ value of 20° [31]. The XG-cl-poly(AAm-co-AA) hydrogel diffractogram shows a peak at a 2θ value of 20.72° for xanthan gum. It was observed that in hydrogel formation crystallinity of the native xanthan gum increases. It was observed that XG-cl-poly(AAm-co-AA) hydrogel displayed eighteen observable and discrete diffraction peaks at 2θ values of 19.23°, 20.72°, 23.13°, 25.18°, 27.22°, 28.02°, 29.27°, 30.24°, 31.58°, 33.71°, 40.49°, 44.22°, 45.29°, 56.42°, 57.84°, 60.15°, 66.20°, 75.10°. These peaks displayed the grafted acrylamide and alginic acid onto crude xanthan gum. This outcome confirms that the optimized grafting happened when acrylamide and alginic acid reacted with xanthan gum and the obtained end product was established to be a highly crosslinked hydrogel. Thus intensification in crystallinity of xanthan gum could be ascribed to the impact made by acrylamide and alginic acid. As the synthesis was carried out with microwave irradiation which further significantly improved the crystallinity of xanthan gum-based hydrogel. A similar upsurge in crystallinity with the subsequent microwave treatment of xanthan gum was also observed by Sharma et.al., (2011) Singh et al. (2009) and Anjum et al. (2015) [32,33,34]. 

**Figure 3:**
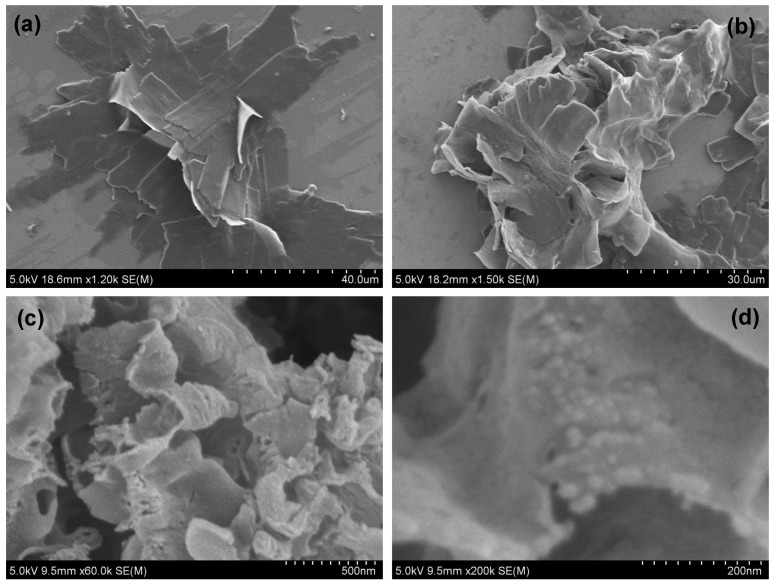
SEM micrographs of xanthan gum-cl-poly(acrylamide-co-alginic acid) hydrogel (**a**–**c**) before adsorption and (**d**) after adsorption.

The SEM images of that XG-cl-poly(AAm-co-AA) hydrogel were displayed in Figure 3. Figure 3a,b depicts the smooth surface of hydrogel whereas Figure 3c displays the highly folded surface with pores at higher magnification. Figure 3d represents the SEM image of XG-cl-poly(AAm-co-AA) hydrogel after adsorption of Cd(II) ions depicting rough surface which may be due to the addition of Cd(II) ions to the surface.

### 2.1. Adsorption of Cadmium Ions by XG-cl-Poly(AAm-co-AA) Hydrogel

#### 2.1.1. Effect of Various Factors

**Figure 4:**
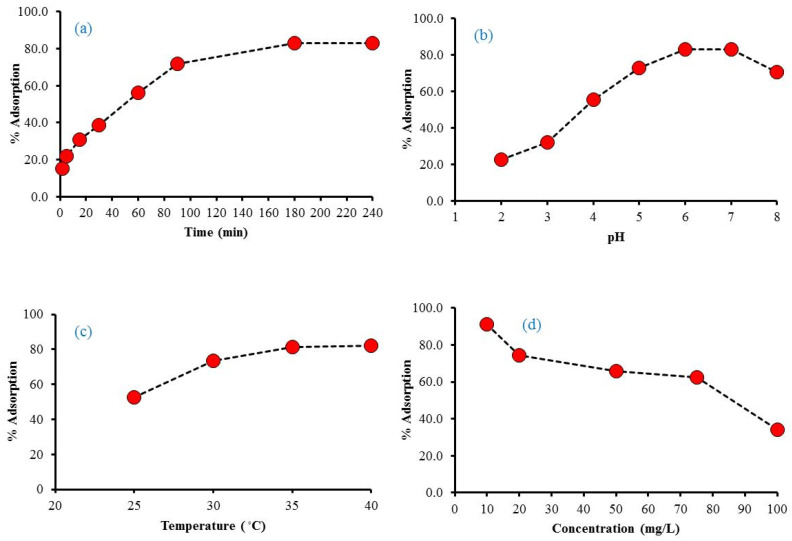
Effect of influential parameters on the adsorption of cadmium ions (**a**) Contact time, (**b**) solution pH, (**c**) temperature, and (**d**) concentration of adsorbate.

Figure 4 shows the effect of various parameters such as contact interval, solution pH, temperature, and concentration of adsorbate on the adsorption of Cd(II) ions onto XG-cl-poly(AAm-co-AA) hydrogel. Figure 4a displays the outcome of contact time on the adsorption rate as an escalation in time duration enhances the probability of interactions between the adsorbate and adsorbent surface. The result shows the initial increase in adsorption rate up to 180 min which is probably due to the high accessibility of free or active adsorbent sites. However, afterward, a nearly constant rate was obtained due to the partial saturation of accessible active sites. 

The effect of solution pH on the adsorption rate is presented in Figure 4b and was studied at different pH values such as 2, 4, 5, 6, 7, and 8. Results show that the rate first increased up to 7 and then decreased to 8, thus, working pH was found to be 7. The % adsorption of the XG-cl-poly(AAm-co-AA) hydrogel first improved with the initial upsurge in pH value because of the ionization of the hydrophilic polymer network. Major functional units existing in the structure of hydrogel are, -NH_2_, COOH, and –OH. The adsorption rate enhanced with the rise in pH because of the ionization of –NH_2_ and –COOH groups at pH greater than the pka value. This favored the intermolecular interactions among the cadmium (II) ions and hydrophilic XG-cl-poly(AAm-co-AA) hydrogel networks.

The influence of temperature on the adsorption rate was also analyzed and considered at four altered temperatures; 25 °C, 30 °C, 35 °C, and 40 °C. The temperature influence generalizes the chemical or physical nature of the adsorption process. Results presented in Figure 4c show the maximum adsorption rate of 84% was obtained at 35 °C suggesting the inclination of Cd(II) ion adsorption towards chemical behavior. Further increase in temperature resulted in a constant adsorption rate indicating the saturation at 35 °C. This study indicated that the temperature played a critical part in the undertaken Cd(II) ions adsorption. 

The significance of Cd(II) ion concentration on the adsorption rate was analyzed in the range of 10–100 mg·L^−1^. The results presented in Figure 4d show the fall in % adsorption with the upsurge in Cd(II) concentration and the maximum rate was obtained at 10 mg·L^−1^. The increase in concentration overloaded the solution with Cd(II) ions which made it difficult for the hydrogel to adsorb the ions, as a result of which the adsorption decreased. Maximum adsorption of 92% was obtained at 10 mg·L^−1^ Cd(II) concentration. 

#### 2.1.2. Adsorption Kinetics 

Adsorption kinetics includes the variation in the adsorption characteristics of the system w.r.t. time, where the amount of surface covered delivers significant insight on the rate of the process. The rating mechanism of Cd(II) ion adsorption onto XG-cl-poly(AAm-co-AA) hydrogel was considered using two kinetic models, pseudo-first-order (PFO) and pseudo-second-order (PSO). These models were applied to the kinetic data obtained for a time at an interval of 5–180 min. The linear form of the models was used and the equations representing them were [35]:

(1) $$\mathrm{Log}\;\left(q_{e}-q_{t}\right)=\mathrm{Log}\;q_{e}-K_{1}t\;\mathrm{Pseudo}-\mathrm{first}-\mathrm{order}$$

(2) $$\frac{t}{q_{t}}=\frac{1}{k_{2}q_{e}{}^{2}}+\frac{t}{q_{e}}\;\mathrm{Pseudo}-\mathrm{second}-\mathrm{order}$$

where q_e_ and q_t_ (mg·g^−1^) denote the Cd (II) ion amount at equilibrium (e) and at time t. k_1_ (min^−1^) and k_2_ (g·mg^−1^·min^−1^) are the pseudo-first-order and pseudo-second-order rate constant. The calculated parameter values of the two models are presented in Table 1. Assessment of the R^2^ values disclosed that the pseudo-first-order model better fitted the kinetic data than that of the pseudo-second-order model. Additionally, the experimental qe value (91.26 mg·g^−1^) was found to best correlate with the calculated qe value from pseudo-first-order (95.9 mg·g^−1^). In addition, the best fit was attained at a higher initial concentration of 60 mg·L^−1^ signifying the reason for its better fit [36]. Undertaken adsorption process signifies that the Cd(II) ions adsorption was quite a tedious process of synchronized action of several reactions, and might encompass various interactions among adsorbent and adsorbate. 

#### 2.1.3. Adsorption Isotherms

**Figure 5:**
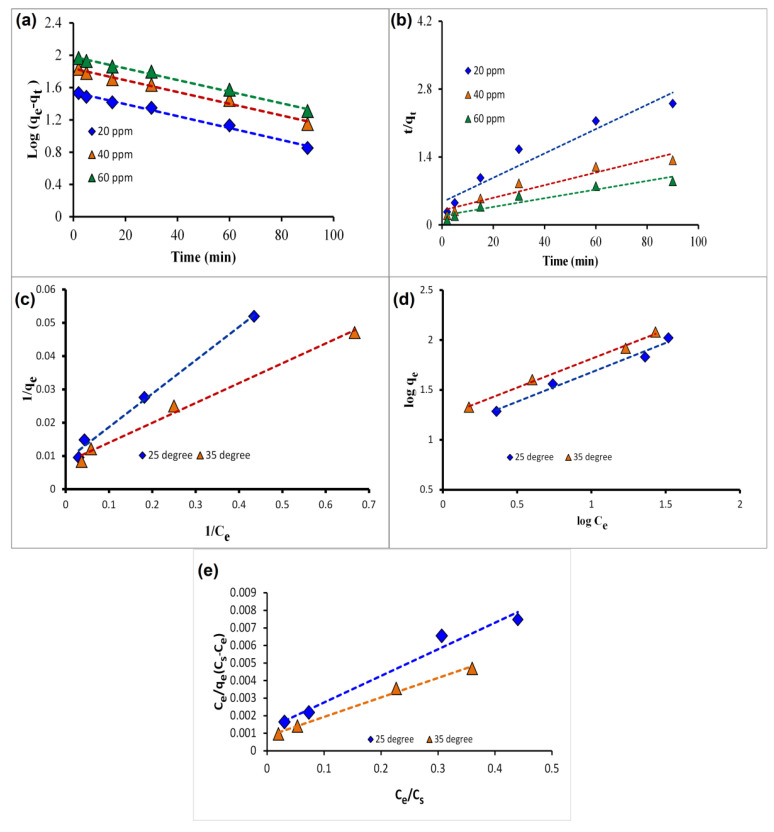
Adsorption kinetic models (**a**) Pseudo-first-order, (**b**) Pseudo-second-order, and Adsorption isotherm models (**c**) Langmuir isotherm, (**d**) Freundlich isotherm and (**e**) BET isotherm.

Figure 5c,d shows the linear Langmuir, and Freundlich isotherm models for the adsorption of Cd(II) ions onto hydrogel at two temperatures, viz., 25 °C and 35 °C. The equations used for the linear isotherm models are [37]:

(3) $$\frac{1}{q_{e}}=\frac{1}{\left({\mathrm{qmK}}_{L}\right)C_{e}}+\frac{1}{\mathrm{qm}}\;\mathrm{Langmuir}$$

(4) $${\mathrm{Log}\;q}_{e}={\mathrm{Log}\;K}_{F}+\frac{1}{n}{\mathrm{Log}\;C}_{e}\;\mathrm{Freundlich}$$

In addition, the multilayer adsorption isotherm model, BET, was also applied to the isotherm data. BET isotherm was first established in 1938 by Brunauer and his coworkers and it is considered as one of the most proficient models for expressing the adsorption phenomena. It helps in determining various parameters of the undertaken adsorption such as heat of adsorption, adsorption capacity, and multilayer adsorption behavior. The linear equation used for the analysis is [38]:

(5) $$\frac{C_{e}}{q_{e}\left(C_{s}-C_{e}\right)}=\frac{1}{q_{s}C_{\mathrm{BET}}}+\frac{\left(C_{\mathrm{BET}}-1\right)}{q_{s}C_{\mathrm{BET}}}\left(\frac{C_{e}}{C_{s}}\right)\;\;\;\mathrm{BET}$$

Here C_e_ and C_s_ denote the equilibrium concentration (mg·L^−1^) and monolayer saturation concentration (mg·L^−1^). C_BET_ and qs represent the BET adsorption isotherm constant (L·mg^−1^) and isotherm saturation capacity (mg·g^−1^), respectively.

The values obtained for different parameters of the three isotherm models are displayed in Table 2. The comparison of three fitted isotherm models by correlation coefficient (R^2^) showed that the Freundlich isotherm fitted the best to the undertaken adsorption experiment at 35 °C. This suggested the Cd(II) ions followed multilayer adsorption onto the XG-cl-poly(AAm-co-AA) hydrogel. Isotherm analysis also suggested that the Cd(II) ions were physically adsorbed onto the surface. Maximum adsorption capacity achieved was 114.94 and 125.00 mg·g^−1^ at 25 °C, and 35 °C, respectively, which was found to be quite high as compared to other adsorbents used for Cd(II) ion adsorption testified in the literature such as 27.3 mg·g^−1^ by commercial activated carbon [39], 2.9 mg·g^−1^ for Fe_3_O_4_/AC [40], 7.4 mg·g^−1^ by magnetic oak bark biochar [41], 17.54 mg·g^−1^ by CuFe_2_O_4_ nano-particles [42], and 63 mg·g^−1^ by EDTA@Fe_3_O_4_/SC nanocomposite [43]. The factor representing the binding affinity (K_L_) was found to be quite favorable suggesting that the adsorption process relied on high binding affinity among the hydrogel and Cd(II) ions. The value of separation factor (R_L_) obtained is less than 1 indicate the favorable shape of the isotherm. The BET model showed temperature-dependent activity in which better fit was obtained at higher temperatures. Furthermore, it can be generalized that the adsorption of Cd(II) ions onto the XG-cl-poly(AAm-co-AA) hydrogel did not follow the monolayer adsorption.

Comparison data are also given in Table 3 that highlighted the superiority of the synthesized XG-cl-poly(AAm-co-AA) hydrogel as adsorbent for Cd(II) ions. Although, the comparison of adsorption capacity of any adsorbent is not possible since it is governed by various influential factors such as composition, reaction conditions, temperature, solution pH, and functionality extent, etc. So, in the present case, we gave a generalized comparison of various hydrogel-based adsorbents used for Cd(II) adsorption. 

#### 2.1.4. Thermodynamic Analysis

Thermodynamic studies were examined at different temperatures, viz., 298 K, 303 K, 308 K and 313 K. Equations employed for determining Gibb’s energy, enthalpy, and entropy change of the undertaken adsorption process were:

(6) $$∆G^{0}=-{\mathrm{RTlnk}}_{C}$$

(7) $${\mathrm{lnk}}_{C}=-\frac{∆H^{0}}{\mathrm{RT}}+\frac{∆S^{0}}{R}$$

where ∆G^0^ was determined from Equation (6) and ∆H^0^ and ∆S^0^ were determined from Equation (7) by the linear plot of ln kC versus 1/T(K). 

The calculated values are depicted in Table 4. Results indicated that Gibb’s free energy became more negative with the increase in temperature and maximum was obtained at 318 K. This generalized the spontaneous nature of the Cd(II) ion adsorption onto XG-cl-poly(AAm-co-AA) hydrogel. Additionally, enthalpy change was found to be +24.5 J·mol^−1^ suggesting the endothermic nature of the adsorption process. This is also in accordance with the isotherm results in which the better fit was obtained at higher temperatures. The entropy change showed a positive variation too.

#### 2.1.5. Reusability of Hydrogel

**Figure 6:**
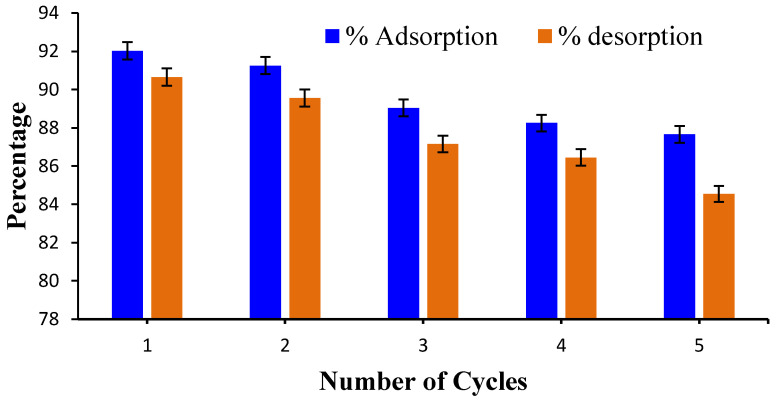
Desorption of Cd(II) ions and reusability of XG-cl-poly(AAm-co-AA) hydrogel for the adsorption.

The commercial-scale applicability of any adsorbent is crucially reliant on its reusable ability. During this study, the adsorbed ions are desorbed from the adsorbent surface to reactivate the adsorption active sites, so that they may possibly be utilized for the next adsorption cycle. The desorption studies of Cd(II) ions were performed in 0.2 M HCl solution. The high concentration of competitive H^+^ ions compete with the Cd(II) ions for the active sites on the XG-cl-poly(AAm-co-AA) hydrogel and will thus, help in the desorption of the adsorbed Cd(II) ions. The results presented in Figure 6 show that a high desorption rate of 91% was obtained during the initial cycle. However, consecutively, the desorption rate decreased to 84% after five cycles. This decrease can possibly be due to the blockage of active sites. Furthermore, the reusability of the synthesized XG-cl-poly(AAm-co-AA) hydrogel was also tested for consecutive five cycles and the rate obtained is depicted in Figure 6. Results showed a decline in adsorption rate from 92% (first cycle) to 86% (after 5th cycle). A likely decline in adsorption rate can be linked to the coverage of some of the active sites by the Cd(II) ions which were difficult to remove from the XG-cl-poly(AAm-co-AA) hydrogel surface. 

## 3. Conclusions

XG-cl-poly(AAm-co-AA) hydrogel is an effective adsorbent for the confiscation of cadmium ions. The FTIR characterization specified the existence of carboxyl, amides, and hydroxyl groups which are recognized as the active sites on the XG-cl-poly(AAm-co-AA) hydrogel for the uptake of cadmium ions from the aqueous system. The kinetic outcomes established that bulk diffusion is the primary mechanism of cadmium ions adsorption. Comparison of the R^2^ values disclosed that the pseudo-first-order model better fitted the kinetic data than that of the pseudo-second-order model. Additionally, the experimental q_e_ value (91.26 mg·g^−1^) was found to best correlate with the calculated q_e_ value from the pseudo-first-order (95.9 mg·g^−1^). The hydrated cadmium ions can merely be adsorbed onto the surface of the XG-cl-poly(AAm-co-AA) hydrogel through physicochemical interactions. Furthermore, the reusability studies demonstrated the usefulness of hydrogel. Fortuitously, all reactants are in the adsorbent are biocompatible, biodegradable, and harmless.

## 4. Materials and Methods

### 4.1. Materials

Xanthan gum, acrylamide and alginic acid were acquired from Sigma-Aldrich, India. Crosslinker N, N-methylene-bis-acrylamide, initiator ammonium persulphate and cadmium nitrate were purchased from Loba Chemie India.

### 4.2. Synthesis of Xanthan Gum-cl-Poly(Acrylamide-co-Alginic Acid)

The 250 mg xanthan gum was dissolved in 100 mL double distilled water. The preparation of XG-cl-poly(AAm-co-AA) hydrogel was carried out with small amendment in procedure stated in our former works. To the above solution of xanthan gum, ammonium persulphate and *N*,*N*-bismethyleneacrylamide were added with uninterrupted stirring at room temperature, the resultant mixture was stirred for 20 min to achieve a homogeneous phase and the accomplished gel was then positioned in microwave oven at 60 W for 1 min. Then, varying amounts of acrylamide and alginic acid (in ratio of 1:1:1; 1:0.5:1; 1:1:0.5) were engrossed into the above gel and stirred for 10 min. This mixture was yet once more placed in microwave oven operated in cyclic mode (on/off) at 60 W, till hydrogel was achieved. The obtained hydrogel was filtered and washed several times to confiscate impurities using distilled water. Finally, hydrogel was freeze-dried. The investigate swelling behavior XG-cl-poly(AAm-co-AA) hydrogel was submerged into distilled water for 24 h to attain maximum swelling. The XG-cl-poly(AAm-co-AA) hydrogel sample with maximum swelling ability was chosen for further investigation. The % swelling was measured by applying the formula discussed in earlier studies [33].

### 4.3. Characterization

FTIR spectrum of XG-cl-poly(AAm-co-AA) hydrogel was recorded using Fourier Transform Infrared Spectrophotometer (Shimadzu IR AFFINITY-I, Japan). The spectrum XG-cl-poly(AAm-co-AA) was measured in the wave number region 4000–400 cm^−1^ by preparing the KBr pellet. The hydrogel samples were prepared using 1.0% KBr pellets and all spectra were recorded with 20 runs per minute at a resolution of 4.0 cm^−1^. The normalization of the peaks was performed by vector normalization method. X-ray pattern of powdered XG-cl-poly(AAm-co-AA) hydrogel was analyzed by X-ray diffractometer with Cu K-α radiation (λ = 1.54 Å) at 45 kV (PAN analytical X′Pert PRO). The surface morphology analysis of dry hydrogel and cadmium ion adsorbed hydrogel were perceived using a model HITACHI S-4800 scanning electron microscope at an accelerating voltage of 25 kV.

### 4.4. Adsorption Experiments

Cadmium nitrate was acquired from Loba Chemie India. Cadmium nitrate was solubilized in deionized water. The cadmium ions adsorption tests were accomplished on a temperature-controlled shaker at 120 rpm. After every test, the suspension was centrifuged to isolate the hydrogel from the aqueous medium. The cadmium ion concentrations of the adsorbate solution were determined using ICP-OES. All the subsequent experiments were comprehended using dosage of 0.5 g·L^−1^ of the XG-cl-poly(AAm-co-AA) hydrogel. First, the pH influence was examined for diverse initial pH values (2, 3, 4, 5, 6, 7 and 8), adjusted with NaOH or HCL. For this, the XG-cl-poly(AAm-co-AA) hydrogel was added to 50 mL of cadmium ions solution (10 mg·L^−1^) and shaken for 180 min at room temperature. Next, effect of time was investigated for the adsorption assays at the time intervals of 20,40, 80, 120, 180 and 240 min with initial cadmium ions concentrations of 10 mg L^−1^, all the flasks were agitated at constant rpm of 120. Similarly influence of four different temperatures, i.e., 25 °C, 30 °C, 35 °C and 40 °C for 180 min, to confirm that the adsorbent/adsorbate system attain the equilibrium. Lastly, kinetic studies were executed, using initial cadmium ions concentrations of 10, 20, and 60 mg·L^−1^ at 298 K, aliquots were withdrawn at diverse times intervals ranging from 0–90 min. Langmuir and Freundlich, models were chosen to analyze the adsorption mechanism. A method suggested in previous studies was used to determine the thermodynamic parameters [52,53,54,55]. To disclose the effectiveness of XG-cl-poly(AAm-co-AA) hydrogel, the reusability was tested by performing numerous adsorption–desorption cycles.

## Figures and Tables

**Table 1:** Adsorption kinetic parameters.

Kinetic Models	Parameters	10 mg·L^−1^	20 mg·L^−1^	60 mg·L^−1^
Pseudo-first-order	q_e_ (mg·g^−1^)	34.8	68.5	95.9
	k_1_ (min^−1^)	1.70 × 10^−2^	1.68 × 10^−2^	1.65 × 10^−2^
	R^2^	0.990	0.986	0.992
Pseudo-second-order	q_e_ (mg·g^−1^)	39.8	76.9	111.1
	k_2_ (g·mg^−1^·min^−1^)	13.4 × 10^−3^	5.6 × 10^−4^	4.2 × 10^−4^
	R^2^	0.929	0.923	0.904

**Table 2:** Adsorption isotherm parameters.

Equilibrium Model	Parameters	25 °C	35 °C
Langmuir isotherm	q_m_ (mg·g^−1^)	114.9	125
	b (L·mg^−1^)	8.3 × 10^−2^	9.1 × 10^−2^
	R_L_	0.37	0.35
	R^2^	0.981	0.990
Freundlich isotherm	K_F_ (L·mg^−1^)	12.21	17.14
	n	1.69	1.72
	R^2^	0.990	0.995
BET	Qs (mg·g^−1^)	75.75	84.74
	C_BET_ (L·mg^−1^)	13.225	14.755
	R^2^	0.963	0.988

**Table 3:** Comparison of monolayer adsorption capacity of XG-cl-poly(AAm-co-AA) hydrogel with other adsorbents reported in the literature for Cd(II) adsorption.

Adsorbent	Adsorption Capacity (q_m_, mg·g^−1^)	References
S(H)-PAA hydrogel	109.8	[44]
SCHBs	95.6	[45]
NNCA hydrogel	9.54	[46]
Bentonite/alginate composite beads	53.2	[47]
Thiol-functionalized mesoporous silica	78	[48]
sodium alginate-meso-2,3-dimercaptosuccinic acid hybrid aerogel	91.2	[49]
β-cyclodextrin-based hydrogel	98.8	[50]
HMO-P(HMAm/HEA) hydrogel	93.86	[51]
**XG-cl-poly(AAm-co-AA) hydrogel**	125	**Present work**

**Table 4:** Thermodynamic parameters for the adsorption of Cd(II) onto XG-cl-poly(AAm-co-AA) hydrogel.

C_o_ (mg·L^−1^)	ΔH^0^ (J·mol^−1^)	ΔS^0^ (J·mol^−1^·K^−1^)	−ΔG^0^ (J·mol^−1^)			
			298 K	303 K	308 K	313 K
20	24.5	0.09	2.32	2.77	3.22	3.67
